# Editorial: Bullying and cyberbullying: their nature and impact on psychological wellbeing

**DOI:** 10.3389/fpsyg.2023.1265031

**Published:** 2023-08-10

**Authors:** Alessandra Fermani, Gonzalo del Moral, Carla Canestrari

**Affiliations:** ^1^Department of Education, Cultural Heritage and Tourism, University of Macerata, Macerata, Italy; ^2^Department of Education and Social Psychology, University Pablo de Olavide, Seville, Spain

**Keywords:** cyberbullying, mockery, violence, mental health, wellbeing

## 1. Introduction

Bullying and cyberbullying are potent forms of violence repeatedly perpetrated by aggressors against victims. They are similar in many facets as both share the same psychological dynamics, comprise a dominion-submission model between the aggressors and the victims, and always present a spectator, even if virtual, to whom the bullies refer. Stereotypical ideas, violated rules, or any feature of a person or group can be the pretext for (cyber)bullying.

If bullying is a type of anti-social behavior that has been studied for decades, cyberbullying is a growing phenomenon. Due to the widespread use of new technologies and the internet, cyberbullying has become even more frequent, especially among young people, who are prone to mobile phone use (Lenhart, 2012; Görzig and Ólafsson, 2013; Shapka et al., 2018). In particular, the social isolation adopted to restrain the COVID-19 pandemic intensified certain elements related to digital sociability (e.g., hyperexposure, diluted public-private-intimate borders, self-spectacularisation) that created conditions exacerbating digital violence and cyberbullying (Hellsten et al., 2021; Martínez-Ferrer et al., 2021).

Cyberbullying victims with low self-esteem and loneliness suffer disorders such as depression, anxiety, suicide ideation, substance abuse, and poor engagement in prosocial behaviors, among others. The adverse impact on a person's wellbeing is significant (Schoeps et al., 2018), and parental attachment plays a crucial role as well (Canestrari et al., 2021). Evidence show that youth reporting low levels of satisfaction with family relationships, negative feelings about school, and lower acceptance levels by their peers were more likely to participate in bullying and cyberbullying (Martínez-Ferrer et al., 2019). This Research Topic aims to deepen one's awareness of the nature of bullying and cyberbullying, including the prevention tools and coping strategies implemented by the various individuals involved in the phenomenon (e.g., violence and aggression, exclusion and superiority, mockery). Psychology has attempted over time to give greater importance to the context according to holistic theories (e.g., social identity theory, social network analysis, correlates theory, personal reputation theory) (Emler and Reicher, 1995), as suggested by Bronfenbrenner's social ecology model. Throughout this general approach, the Research Topic brought together current perspectives on bullying and cyberbullying at various developmental stages, their causes and consequences on different life domains, new evaluation methods in future studies, and training programmes that combat this negative dynamic from a multidisciplinary perspective.

## 2. Papers of the Research Topic

There are 10 manuscripts on this Research Topic. Given the prevalence of modern technologies, this topic is expectedly covered in many studies specifically focused on cyberbullying. Bochaver's opinion article and Shi and Wang's and León-Moreno et al.'s research papers highlighted school bullying, defined as a form of bullying perpetrated by (a) student(s) against (an)other student(s). Bochaver reflects on the complexity of the phenomenon, which, on one side, provokes negative outcomes, and on the other side, serves as a coping strategy for a community, given its realization of psychological needs such as establishing a social hierarchy, reducing emotional tension, and controlling members. Shi and Wang's study on 3,363 middle/high school students reveals a positive relationship between school victimization and Internet addiction, mediated by life satisfaction and loneliness. León-Moreno et al. highlighted the guilt and loneliness experienced in adolescent peer victimization. The study, carried out on a sample of students, shows that adolescents with greater propensity for guilt feel responsible for being victims of peer aggression and for feeling lonely.

Sorrentino et al. and Gao et al.'s studies explore risk factors of cyberbullying and cybervictimisation. In particular, Sorrentino et al. analyzed a sample of students in a year-long longitudinal study and found onset risk factors for cyberbullying (i.e., being male, being involved in school bullying, having low levels of awareness of online risks, and having high levels of affective empathy) for cybervictimisation (i.e., being male, being involved in school bullying and victimization, having high levels of affective empathy and moral disengagement). On the other hand, Gao et al. examined how family incivility, defined as problematic family interactions and parental neglect, impacts cyberbullying perpetration in a sample of university students. They found that family incivility is positively correlated with cyberbullying perpetration, which is influenced by negative emotions, particularly for highly neurotic students.

Moral disengagement in cyberbullying has been highlighted in research by Mateus Francisco et al. and Zhu et al.. Mateus Francisco et al. identified the relationship between moral disengagement and empathy in cyberbullying situations among adolescents. They developed and validated the Empathy Quotient in Virtual Contexts for Portuguese adolescents communicating online and the Process Moral Disengagement in Cyberbullying Inventory (PMDCI) to assess moral disengagement in online communication. Zhu et al. explored the use of aggressive humor as a tool for cyberbullying perpetration. The study, conducted on a sample of university students, revealed that moral disengagement mediates the relationship between cyberbullying perpetration and aggressive humor, which positively relates to moral disengagement, and that moral disengagement is positively related to cyberbullying perpetration.

Violence is the main category underpinning bullying and cyberbullying. Reyes-Martínez et al. studied several forms of violence. The study, which involved adult respondents, revealed that victims relying on cultural activities had higher levels of subjective wellbeing, suggesting that such activities help in coping and adapting to stressful and traumatic situations.

The articles summarized so far focus mainly on victimization, whereas Horink et al.'s research emphasizes *gluckachmerz*, i.e., a feeling of displeasure at others' success, as a potential psychological factor that may trigger aggressive and negative online messages and word of mouth. Finally, Hendry et al. have conducted interviews and focus groups with stakeholders having professional knowledge about cyberbullying to ascertain the principles on which basis cyberbullying prevention and intervention programs can be projected.

## Author contributions

AF: Conceptualization, Resources, Supervision, Writing—original draft. GM: Conceptualization, Resources, Supervision, Writing—original draft, Writing—review and editing. CC: Conceptualization, Project administration, Resources, Supervision, Writing—original draft, Writing—review and editing.
